# Ether-Oxygen Containing Electrospun Microfibrous and Sub-Microfibrous Scaffolds Based on Poly(butylene 1,4-cyclohexanedicarboxylate) for Skeletal Muscle Tissue Engineering

**DOI:** 10.3390/ijms19103212

**Published:** 2018-10-17

**Authors:** Nora Bloise, Emanuele Berardi, Chiara Gualandi, Elisa Zaghi, Matteo Gigli, Robin Duelen, Gabriele Ceccarelli, Emanuela Elsa Cortesi, Domiziana Costamagna, Giovanna Bruni, Nadia Lotti, Maria Letizia Focarete, Livia Visai, Maurilio Sampaolesi

**Affiliations:** 1 Department of Molecular Medicine, Center for Health Technologies (CHT), INSTM UdR of Pavia, University of Pavia, 27100 Pavia, Italy; nora.bloise@unipv.it (N.B.); emanuelaelsa.cortesi@kuleuven.be (E.E.C.); 2 Department of Occupational Medicine, Toxicology and Environmental Risks, ICS Maugeri, IRCCS, 27100 Pavia, Italy; 3 Translational Cardiomyology Laboratory, Department of Development and Regeneration, KUL University of Leuven, 3000 Leuven, Belgium; emanuele.berardi@kuleuven.vib.be (E.B.); robin.duelen@kuleuven.be (R.D.); domiziana.costamagna@kuleuven.be (D.C.); 4 Department of Chemistry “G. Ciamician” and INSTM UdR of Bologna, University of Bologna, via Selmi 2, 40126 Bologna, Italy; c.gualandi@unibo.it (C.G.); marialetizia.focarete@unibo.it (M.L.F.); 5 Health Sciences and Technologies and Interdepartmental Center for Industrial Research (HST-ICIR), University of Bologna, via Tolara di Sopra 41/E, Ozzano dell’Emilia, 40064 Bologna, Italy; 6 Unit of Clinical and Experimental Immunology, Humanitas Clinical and Research Center, 20089 Rozzano, Italy; elisa.zaghi@humanitasreasearch.it; 7 Department of Chemical Science and Technologies, University of Rome Tor Vergata Via della ricerca Scientifica 1, 00133 Roma, Italy; matteo.gigli@uniroma2.it; 8 Human Anatomy Unit, Department of Public Health, Experimental and Forensic Medicine, Center for Health Technologies (CHT), Interuniversity Institute of Myology (IIM), University of Pavia, 27100 Pavia, Italy; gabriele.ceccarelli@unipv.it; 9 Department of Chemistry, Section of Physical Chemistry, University of Pavia, 27100 Pavia, Italy; giovanna.bruni@unipv.it; 10 Civil, Chemical, Environmental and Materials Engineering Department, University of Bologna, Via Terracini 28, 40131 Bologna, Italy; nadia.lotti@unibo.it

**Keywords:** muscle tissue engineering, myogenesis, electrospinning, microfibres and sub-microfibres, biodegradable polyesters

## Abstract

We report the study of novel biodegradable electrospun scaffolds from poly(butylene 1,4-cyclohexandicarboxylate-*co*-triethylene cyclohexanedicarboxylate) (P(BCE-*co*-TECE)) as support for in vitro and in vivo muscle tissue regeneration. We demonstrate that chemical composition, i.e., the amount of TECE co-units (constituted of polyethylene glycol-like moieties), and fibre morphology, i.e., aligned microfibrous or sub-microfibrous scaffolds, are crucial in determining the material biocompatibility. Indeed, the presence of ether linkages influences surface wettability, mechanical properties, hydrolytic degradation rate, and density of cell anchoring points of the studied materials. On the other hand, electrospun scaffolds improve cell adhesion, proliferation, and differentiation by favouring cell alignment along fibre direction (fibre morphology), also allowing for better cell infiltration and oxygen and nutrient diffusion (fibre size). Overall, C2C12 myogenic cells highly differentiated into mature myotubes when cultured on microfibres realised with the copolymer richest in TECE co-units (micro-P73 mat). Lastly, when transplanted in the tibialis anterior muscles of healthy, injured, or dystrophic mice, micro-P73 mat appeared highly vascularised, colonised by murine cells and perfectly integrated with host muscles, thus confirming the suitability of P(BCE-*co*-TECE) scaffolds as substrates for skeletal muscle tissue engineering.

## 1. Introduction

Musculoskeletal diseases (MSDs) represent a common cause of disability, often resulting in degeneration and/or loss of structure and function of the skeletal muscle tissue [1,2]. MSDs include rheumatoid arthritis, osteoarthritis, tendinitis, fibromyalgia, and bone fractures and the risk of developing these disorders increases with age. Although advancing in reconstructive surgical techniques, the outcomes can be unsatisfactory in treating more severe or chronic skeletal muscle injuries [3]. Tissue engineering may offer innovative biomaterials systems owing to attractive features for skeletal muscle tissue regeneration. For instance, new materials can be tailored to degrade at defined rates in vivo [4], interact with the host immune system [5], provide cells with physiologically relevant mechanical and chemical properties [6], modulate cell behaviour via defined topographical features [7], and elicit favourable gene expression by delivering external stimuli to seeded cells [8]. Over the past few years, considerable efforts have been made to meet these requisites using a variety of methods and biomaterials [9]. Several techniques have been adopted to fabricate artificial scaffolds mimicking the natural extracellular matrix (ECM) such as electrospinning, self-assembly, phase separation, and drop casting [10,11,12]. Among these techniques, electrospinning is particularly interesting since it enables the production of fibres that reach the size scale of natural ECMs, it is scalable, and can process various natural and synthetic polymers into fibres with controlled dimensions and orientation [13,14]. These characteristics make electrospinning a useful technique to assembly fibrous scaffolds for tissue engineering applications [15,16]. Electrospun aligned fibres mimic the anisotropic structural organization of elongated myofibres in skeletal muscle and then can provide vital cues for morphogenesis [17]. A number of polymeric biomaterials have been manufactured by electrospinning [18,19,20] and applied as potential artificial scaffolds for skeletal muscle regeneration [21,22].

Biodegradable polymers and nanomaterials are commonly employed for many biomedical and pharmaceutical applications [23]. In tissue engineering, a wide number of natural (e.g., collagen) and synthetic polymers have been used as scaffolds for replace or repair injured and diseased organs [24]. Over naturally derived materials, synthetic scaffolds offer certain advantages in that they can be precisely characterised and fabricated with great control over physical and chemical properties [24,25]. To date, because of the relative ease of their synthesis among the degradable biomaterials, aliphatic polyesters represent the most developed class [20,26,27]. This aspect is mainly due to their appreciated mechanical properties, as well as the commercial availability [4]. Polyester substrates, such as poly(lactic acid) (PLA), poly(glycolic acid) (PGA), poly(ε-caprolactone) (PCL), and their copolymers have been approved by the Food and Drug Administration (FDA) [28,29]. Several products based on these polymers are suitable for a wide range of applications including sutures, bone screws, implants and, more recently, delivery systems and tissue engineering scaffolds [30]. However, the difficulty to tailor solid-state properties to fulfil every need hampers their full exploitation. Poly(butylene 1,4-cyclohexanedicarboxylate) (PBCE) may represent a valid alternative to the aforementioned aliphatic polyesters for the fabrication of tissue engineering scaffolds. The presence of an aliphatic ring along the polymer backbone confers to PBCE a good thermal stability and interesting mechanical properties. On the other hand, the high crystallinity degree and chain rigidity of PBCE limit its use, particularly in those domains where fast degradation rate and improved chain flexibility are required [31]. A strategy to help overcome these disadvantages is copolymerisation [32]. Random copolymers of PBCE containing different amount of polyethylene glycol (PEG) -like moieties (TECE), namely P(BCE-*co*-TECE) copolymers, have been synthesised for food packaging applications [32]. Compared to PBCE homopolymer, they display remarkably improved chain flexibility and biodegradability.

A recent study demonstrated that the presence of ether-oxygen atoms in poly(butylene succinate)-based scaffolds, coupled with fibre alignment, promoted the biological interaction, favouring cell attachment and osteogenic differentiation [33]. In view of the above-mentioned outcomes, in this study we investigated, for the first time, the impact of the chemical composition, i.e. the amount of TECE co-units along PBCE backbone, on tissue regeneration. In particular, aligned microfibrous and sub-microfibrous matrices composed of P(BCE-*co*-TECE) random copolymers were fabricated and tested for skeletal muscle tissue engineering purposes. Morphological, physical, and mechanical properties of the PBCE and P(BCE-*co*-TECE) fibrous scaffolds were explored in detail. The myogenic potential of myoblasts cultured on these surfaces was tested, while implantation of these scaffolds in healthy, freeze-injured, and dystrophic murine muscles was performed to analyse the biocompatibility of P(BCE-*co*-TECE) in both physiological and pathological conditions, including acute and chronic muscle degeneration.

## 2. Results

### 2.1. Physical and Mechanical Characterisation of Electrospun Scaffolds

**Figure 1:**
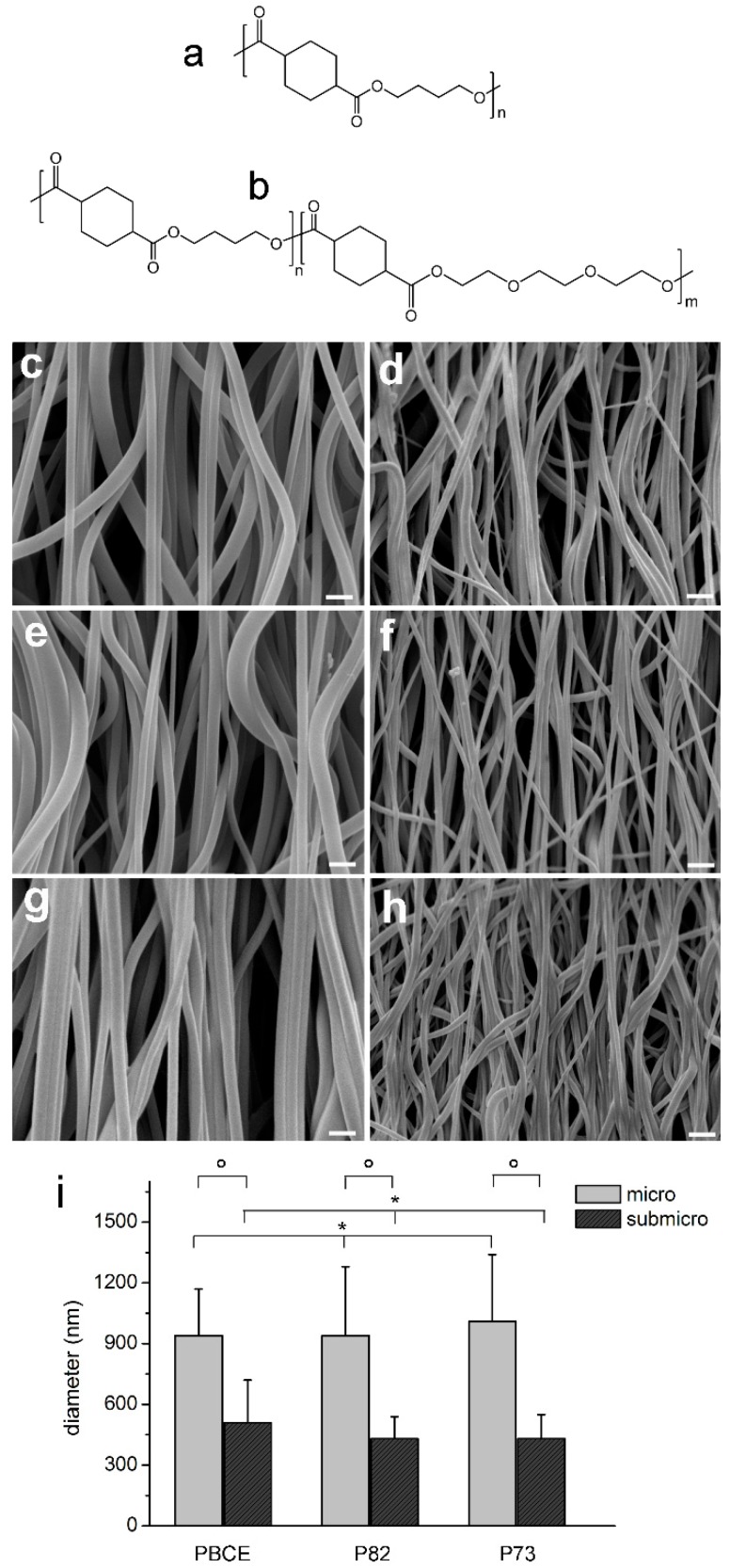
Poly(butylene 1,4-cyclohexanedicarboxylate) homopolymer (PBCE) and poly(butylene 1,4-cyclohexandicarboxylate-*co*-triethylene cyclohexanedicarboxylate) (P(BCE-*co*-TECE)) scaffolds. Chemical structures of (**a**) PBCE homopolymer and (**b**) P(BCE-*co*-TECE) copolymers. SEM micrographs of (**c**) micro-PBCE, (**d**) sub-micro-PBCE, (**e**) micro-P(BCE80-*co*-TECE20) (P82), (**f**) sub-micro-P82, (**g**) micro-P(BCE70-*co*-TECE30) (P73), and (**h**) sub-micro-P73. Scale bars = 2 µm. (**i**) Fibre diameters; * *p* > 0.05; ° *p* < 0.001.

PBCE, P73, and P82 polymers were processed through electrospinning to obtain six electrospun scaffolds made of aligned fibres: micro-PBCE and sub-micro-PBCE, micro P82 and sub-micro-P82, and micro-P73 and sub-micro-P73. The electrospinning conditions, as reported in Supplementary Table S1, are the result of a set of experiments aimed at optimising electrospinning parameters to obtain bead-free sub-micrometric and micrometric fibres for each polymer. 2,2,2-Trifluoroethanol was chosen as a solvent for the electrospinning process because (i) it quickly dissolves all the investigated polymers and (ii) it is suitable for electrospinning process having a high relative dielectric constant (ε_r_~28) and relatively low boiling point (T_b_~74 °C). The control of fibre diameter was achieved by varying polymer solution concentration. Panels in Figure 1c–h show SEM images of electrospun scaffolds, while the corresponding fibre diameter distributions are shown in Figure 1i. In particular mean diameter values were (940 ± 230) nm for micro-PBCE, (510 ± 210) nm for sub-micro-PBCE, (940 ± 340) nm for micro P82, (430 ± 110) for sub-micro-P82, (1010 ± 330) nm for micro-P73, and (430 ± 120) nm for sub-micro-P73.

Calorimetric data of films and scaffolds measured by Differential Scanning Calorimetry are reported in Supplementary Table S3. For all polymers, crystallinity degrees (χ_c_) were found to be higher for the films compared to electrospun scaffolds. Furthermore, PBCE samples are highly crystalline with broad melting endotherm peaks, while for P(BCE-*co*-TECE) copolymers *T_m_* and Δ*H_m_* decreased with the increase of TECE content (Supplementary Table S3).

Static contact angle measurements have been conducted on films obtained by hot-pressing to evaluate polymer surface wettability. By the introduction of an increasing amount of TECE co-units along PBCE backbone, an enhancement of the hydrophilicity of the material was observed: water contact angle value changed from 96° ± 2° to 88° ± 4° and 82° ± 2° for PBCE, P82 and P73, respectively.

**Figure 2:**
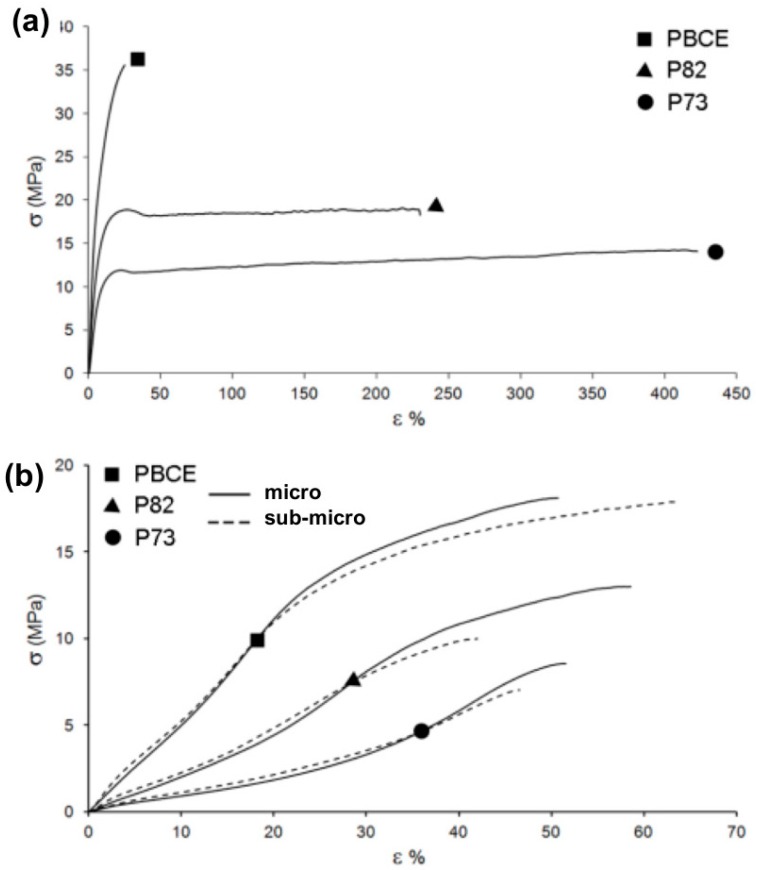
Substrate mechanical properties. Representative stress–strain curves of PBCE (square), P82 (triangle), and P73 (circle): (**a**) films and (**b**) electrospun scaffolds with micrometric fibres (solid line) and sub-micrometric fibres (dashed line).

Also, the tensile behaviour of the investigated polymers (Figure 2 and Supplementary Table S4) was dependent on the chemical composition. PBCE displayed the highest elastic modulus, and it was the stiffest material among the synthesised polyesters with a relatively low deformation at break. On the other hand, the presence of an increasing amount of TECE co-units caused a regular decrease of the elastic modulus and a significant improvement of the stress at break. Furthermore, scaffolds were characterised by a ca. 10× lower elastic modulus and were less strong compared with the corresponding film specimens. No significant difference in stress–strain behaviour was detected between micrometric and sub-micrometric electrospun fibres (Supplementary Table S4).

The hydrolysis profile of the synthesised polymers in physiological environment was evaluated by measuring the residual mass and molecular weight of films immersed in phosphate buffer saline (PBS) at 37 °C for a time lapse between few days and seven months. In this time interval, no significant weight loss was measured for the tested polymers, which also maintained their integrity over time. On the other hand, all samples underwent a decrease of residual number average molecular weight (M_n-res_%) with time (Supplementary Figure S1). The decrease of M_n_ with time, thus the rate of ester cleavage, was higher with the increase of TECE amount. However, after 200 days the maximum decrement was about 30% (P73) and it did not determine the formation of chains short enough to be soluble in water that, in turn, are responsible of sample weight loss.

### 2.2. In Vitro Studies of Myogenic Potential

#### 2.2.1. C2C12 Cell Proliferation Assays

**Figure 3:**
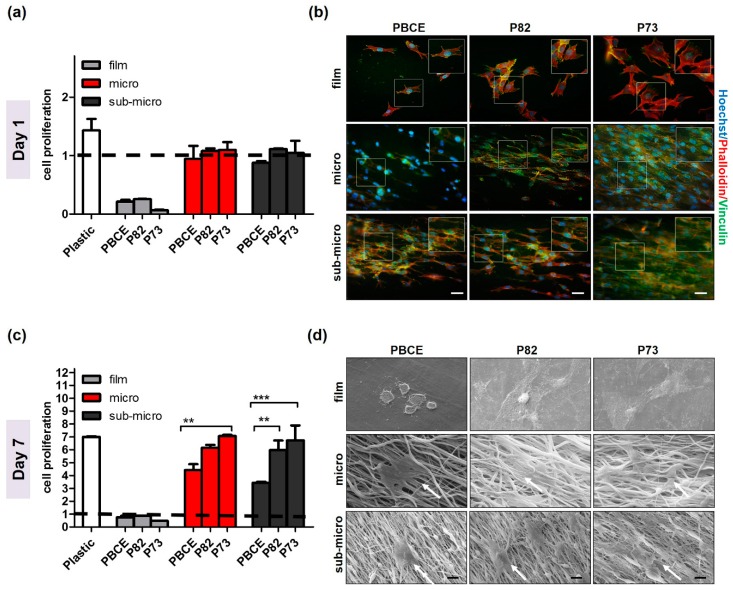
C2C12 proliferation and morphology on microfibrous and sub-microfibrous PBCE and P(BCE-*co*-TECE) scaffolds. (**a**,**c**) Cell growth evaluation was performed using the CCK8 assay at day 1 (**a**) and 7 (**c**) of culture. Cell proliferation (fold increase) is plotted as the ratio between number of cells at each time and number of cells seeded (1.0 × 10^5^ cells/scaffold) on day 0 (indicated by dashed line); ** *p* < 0.01; *** *p* < 0.001. (**b**) Expression of focal adhesion protein vinculin (green). The cytoskeleton organisation was observed by F-actin staining with Phalloydin (red). Nuclei were stained with Hoechst 33342 (blue). Magnified areas of cells are shown in insets. Scale bars = 50 μm. (**d**) Representative scanning electron microscopic images of the cells cultured on PBCE, P82, and P73 materials. Scale bars: 10 µm. White arrows are positioned to indicate C2C12 cells.

Studies of myoblasts proliferation on P(BCE-*co*-TECE) scaffolds have been performed as indicated in Table 1. Specifically, C2C12 cell proliferation rate, defined as the increase in the ratio of cell number at day 1 and day 7 over the seeded cell number, was determined (Figure 3a,c). At day 1, the fold increase value of cell cultured in all electrospun surfaces was approximately 1, which was higher than film counterparts (~0.2) (Figure 3a). Phalloidin and vinculin staining confirmed these proliferation rates (Figure 3b). Once attached to the films, C2C12 showed a round-shape morphology, whereas when cultured on the electrospun substrates they were able to spread across the scaffold surface extending their processes and developing F-actin fibres in the direction of the underlying fibres. The abundance of focal adhesion vinculin confirmed the established strong functional adhesion of cells to the substrates (Figure 3b). At day 7 of cell culture, significant increase of cell proliferation was observed on electrospun surfaces (nearly 4.0, 6.0, and 7.0 on PBCE, P82, and on P73, respectively, for both fibres dimensions) (Figure 3c). Statistical differences of C2C12 proliferation rate among the electrospun surfaces showed a lower cell growth on PBCE when compared to P82 scaffolds (*p* < 0.01 between sub-microfibres structure) and P73 (*p* < 0.01 between microfibres, and *p* < 0.001, among sub-microfibres structures). Cell cultures exhibited a higher growth on P73 than P82 mats, as well on microfibres and sub-microfibres meshes of all electrospun mats as compared to their control films (Figure 3c). These observations were also confirmed by SEM analysis at 7 days that showed the presence of the cells into the fibrous structures, organised in layers parallel to the axes of the underlying fibres (Figure 3d).

#### 2.2.2. C2C12 Differentiation Assays

**Figure 4:**
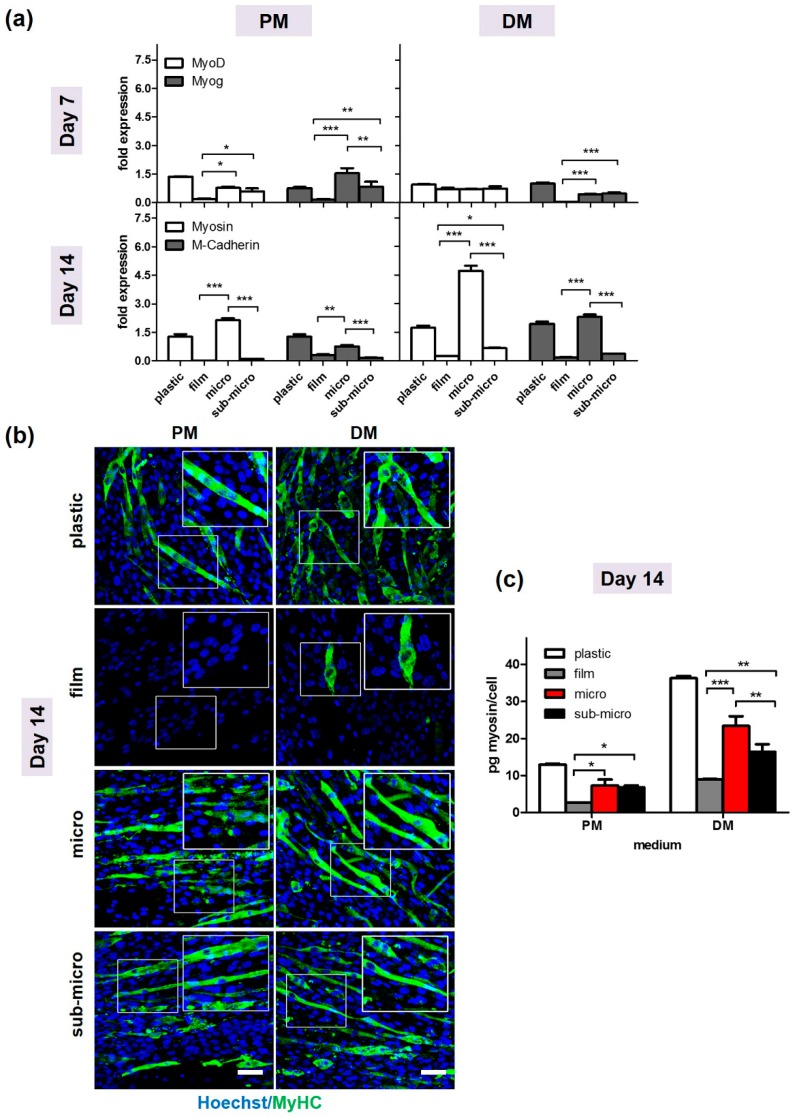
Differentiation of C2C12 cells on electrospun P73 scaffolds.C2C12 cells were seeded on film, micro-, and sub-micro-P73 scaffolds and cultured in proliferative medium (PM) or differentiation medium (DM) for 7 and 14 days, respectively. (**a**) By qRT-PCR, MyoD, and Myog gene expression levels were analysed at day 7 whereas MyHC and M-cadherin at day 14 in PM or DM. Values were normalised against phosphoglycerate kinase (PGK) expression. (**b**) Immunofluorescence images of cells cultured for day 14 in PM and DM. MyHC is stained in green (Alexa Fluor 488) and nuclei are stained in blue (Hoechst 33342). Magnified areas of myotubes in insets. Scale bars: 50 μm. (**c**) MyHC protein quantification by ELISA assay at day 14 in PM or DM; * *p* < 0.05; ** *p* < 0.01; *** *p* < 0.001. Plastic = standard tissue culture plates; PM = Proliferation Medium; DM = Differentiation Medium.

Next, we investigated the myogenic differentiation potential of myoblasts cultured on P73 scaffold, which showed the best proliferation rate for both fibres dimensions. Specifically, C2C12 cells were seeded on film, microfibrous and sub-microfibrous P73 scaffolds and incubated in proliferation (PM) or differentiation (DM) media for 7 and 14 days. Changes of myogenic potential were analysed by Quantitative Reverse Transcription Polymerase Chain Reaction (RT-qPCR) (Figure 4a), immunofluorescence (Figure 4b) and ELISA assays (Figure 4c). MyoD was overexpressed in cells cultured on both microfibrous and sub-microfibrous compared to those cultured on film in proliferative conditions (*p* < 0.05) (Figure 4a). Myogenin (Myog) at day 7, showed higher expression in cells cultured on microfibrous and sub-microfibrous compared to those cultured on film surfaces in both PM (*p* < 0.001 and *p* < 0.01, respectively) and DM media (*p* < 0.001) (Figure 4a).

MyHC and M-cadherin were highly expressed in C2C12 cells cultured on micro-P73 scaffold, in both PM and DM media (*p* < 0.001) (Figure 4a). MyHC expression was also significantly higher in C2C12 cultured on micro-P73 than those cultured on plastic tissue culture plates at day 14 in DM conditions (*p* < 0.001) (Figure 4a).

Immunofluorescence experiments for myogenic protein localisation showed consistent results with gene expression analysis in the culture conditions adopted (Figure 4b). Specifically, MyHC signal, which identifies the terminally-differentiated myotubes, was not detectable on C2C12 cells cultured on film substrate in both PM and DM media, indicating an impairment of myogenic differentiation (Figure 4b). In contrast, MyHC localisation was observed in cells cultured on microfibrous and sub-microsubstrates (Figure 4b). Moreover, MyHC protein levels were quantified by ELISA assay on total protein extracts at day 14 form myogenic induction and the results further confirmed the immunofluorescence analysis (Figure 4c). Indeed, a higher MyHC content was measured in cells cultured on both micro- and sub-micro P73 if compared to those cultured on film (Figure 4c).

### 2.3. In Vivo Electrospun Scaffolds Biocompatibility

#### 2.3.1. Muscle Implantation with the P73 Scaffold

**Figure 5:**
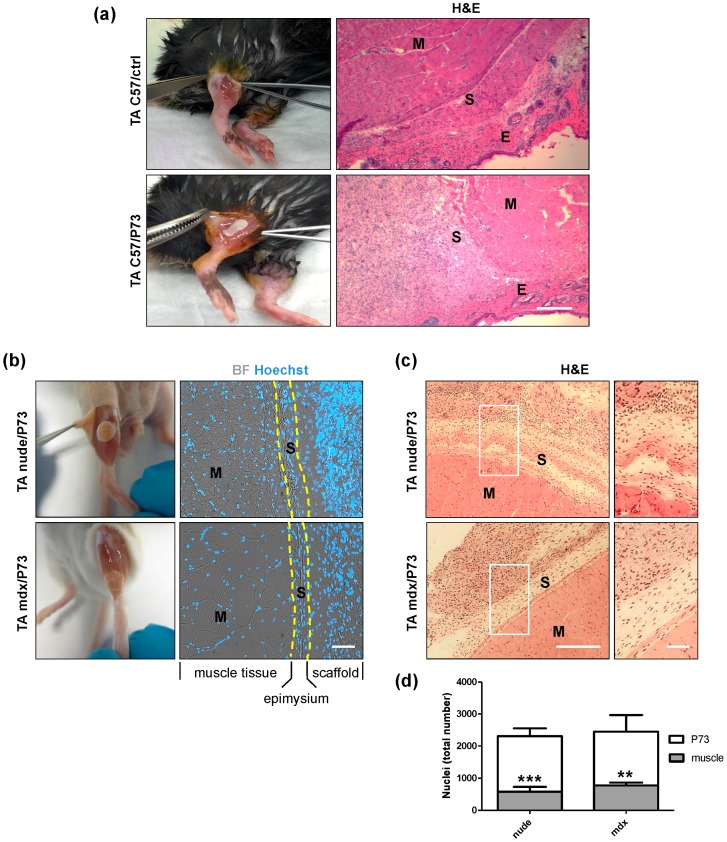
Histological analysis of P73-implanted muscles. (**a**) Exposed tibialis anterior (TA) muscles and H&E staining performed on TA muscles collected from C57BL/6 wt mice without implant (upper panel) and 4 weeks after P73 implantation (lower panel). Micrographs show the presence of the scaffold, indicated with letter S, among the muscle (M) and the epidermis (E). (**b**) Exposed TA muscles showing the micro P73-implanted regions from nude (upper panel) and *mdx* (lower panel) mice 6 weeks after P73 implantation. In TA muscles from C57BL/6 wt (**a**), nude (**b**) and *mdx* (**b**) mice, P73 scaffold adheres to the epimysium of host muscle tissues. Dashed yellow lines indicate the borders among muscle, epimysium and scaffold tissues. Blu = nuclei; grey = bright field. (**c**) Scaffolds appeared highly cellularised, if compared with the muscle tissue of implanted area, as shown by H&E staining. Similar amounts of cell-colonised scaffold were found between C57BL/6 wt (**a**), nude (**c**) and *mdx* (**c**) muscles. Upper right and lower right panels in (**c**) show magnification areas of representative regions of rectangles from nude and mdx muscles, respectively. (**d**) Quantitative analysis of (**c**) (nude mice *n* = 5, mdx mice *n* = 3); ** *p* < 0.01; *** *p* < 0.001 vs. P73. Scale bars: a = 100 µm b = 50 µm; c = 100 µm; c (magnification) = 50 µm.

**Figure 6:**
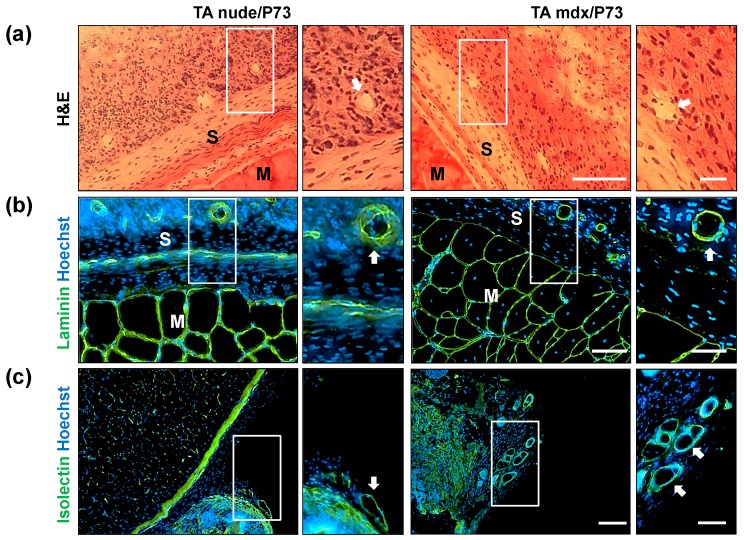
Vascularisation analysis of P73-implanted scaffolds. (**a**) H&E staining was performed on tissue collected from nude and *mdx* mice 6 weeks after P73 implantation. Immunofluorescence analysis (**b**,**c**) for laminin (green, **b**) and isolectin (green, **c**) showed the presence of capillaries and large vessels within the implanted scaffolds from nude and *mdx* muscles. Magnification areas (right panels) show detailed structures of vessels (white arrows), from rectangles. Nuclei are stained in blue with Hoechst. Scale bars: left panel **a** = 100 µm; right panel **a** = 25 µm; left panel **b** = 50 µm right panel **b** = 25 µm; left panel **c** = 100 µm; right panel **c** = 50 µm.

**Figure 7:**
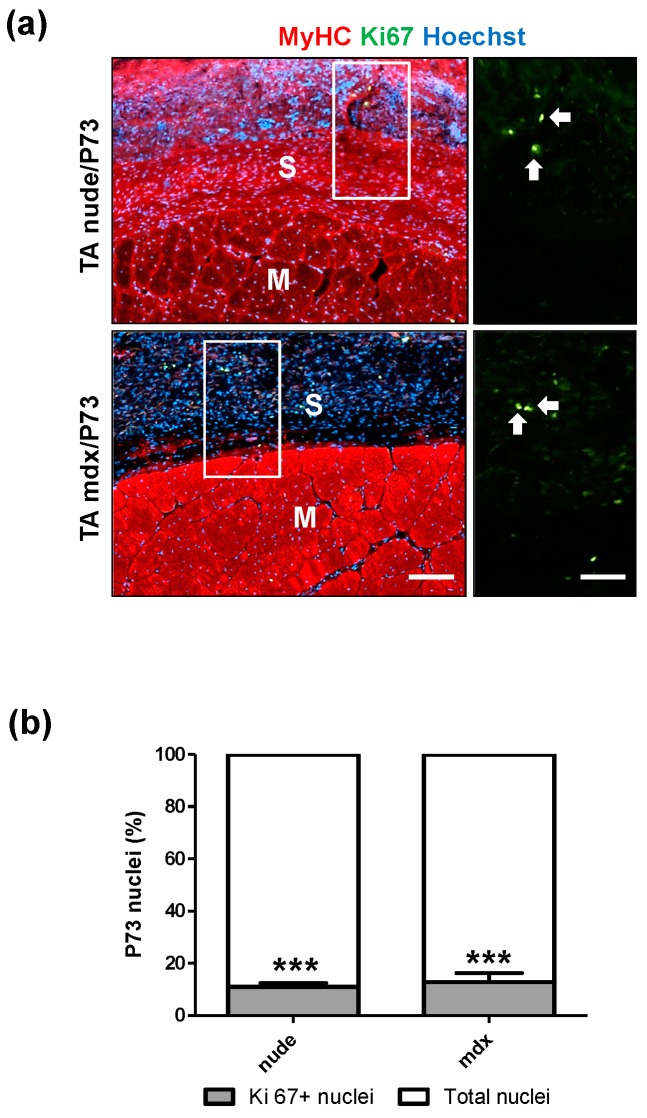
Ki67 positive cells populating P73-implanted scaffold. (**a**) Ki67 staining in P73-implanted tibialis anterior (TA) muscles from nude and *mdx* mice. (**b**) Quantitative analysis of Ki67 positive cells (green) reveals that only ~10% of total cells colonisingP73 scaffold from both nude and *mdx* implanted muscles are Ki67 positive (nude mice *n* = 3, *mdx* mice *n* = 3). Magnification areas (upper right and lower right panels) from rectangles show Ki67 positive nuclei (white arrows). MyHC signal is in red while nuclei are stained in blue with Hoechst. *** *p* < 0.001 vs. total nuclei. Scale bars: lower left panel = 100 µm; lower right panel = 25 µm.

Since in vitro assays performed on all the scaffolds produced identified P73-micro as the best synthetic support for cell growth and differentiation, we further investigated its biocompatibility by in vivo transplantation studies. In particular, tibialis anterior (TA) muscles of C57BL/6 wt (Figure 5a) and *mdx* dystrophic mice (Figure 5b) were implanted with P73 scaffolds and analysed 4 and 6 weeks after implantation, respectively (Supplementary Figure S2a). In addition, athymic nude mice (Figure 5b and Supplementary Figure S2) were implanted with P73 scaffolds three days after acute muscle damage (e.g., freeze-injury) and analysed 6 weeks later (Supplementary Figure S2a). In all mice analysed no migration of scaffolds from the transplanted site was observed (Figure 5a,b) and, in accordance with the analyses of physical characterisation (Supplementary Table S4; Figure 1 and Figure 2), no degradation of scaffolds was detected. The number of neuromuscular junctions near to the transplanted area was not altered in P73-implanted muscles compared to controls (Supplementary Figure S5a,b). Histological analysis of transplanted regions showed a complete integration of P73 scaffolds with the host tissues (Figure 5a–c). Specifically, 4 and 6 weeks after transplantation, P73 appeared highly cellularised and anatomically integrated with the epimysium of the host muscle tissue to the same extent in all models analysed (Figure 5a–c). H&E staining revealed a higher number of nuclei in the scaffolds implanted in muscles from nude and *mdx* mice compared with the area of muscle tissue underneath the implants (*p* < 0.001 and *p* < 0.01, respectively) (Figure 5c,d). Furthermore, we found that at least part of the cells populating the implanted scaffolds is of endothelial origin, as shown by H&E (Figure 6a), and laminin staining (Figure 6b). These evidences demonstrated that the histological integration of P73 was also mediated by vascularisation processes both at the edge of the contact site between the scaffold and the epimysium (Figure 6a,b) and along the external borders of the scaffolds as showed by isolectin staining (Figure 6c). Immunofluorescence analysis for von Willebrand factor and α-sma in P73-implanted muscles from C57BL/6 healthy mice, confirmed the presence of vessels also in the scaffolds of not injured muscles (Supplementary Figure S3). Next, we found that only ~10% of the total cells populating the scaffolds were Ki67positive in implanted muscles of both nude and *mdx* mice (*p* < 0.001), showing that the presence of the scaffold per se did not induce abnormal proliferation in the host muscle tissue (Figure 7a,b).

#### 2.3.2. In Vivo Local Inflammation Analysis of Implanted-Scaffold

**Figure 8:**
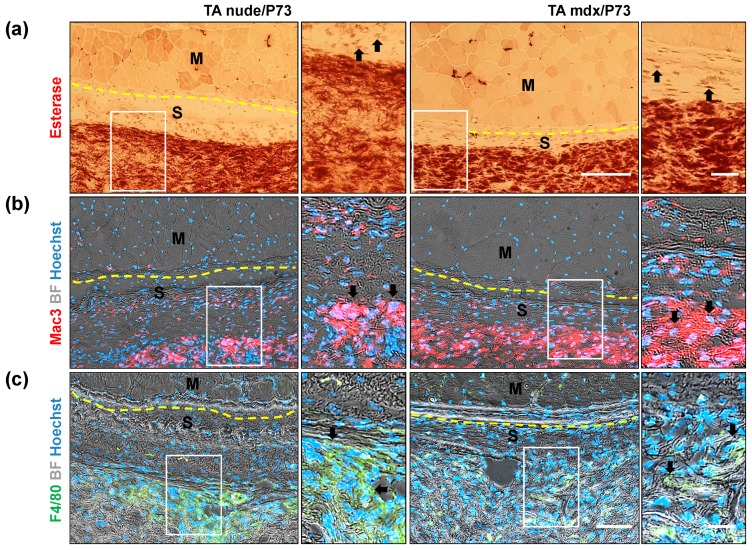
Analysis of inflammatory cells colonisingP73 tissues. Representative images of nonspecific esterase (for macrophages) and immunofluorescence staining of serial muscle sections from P73 tibialis anterior(TA) implanted muscles reveal that the majority of the cells populating scaffolds come from inflammatory origin, as showed by esterase (brown, **a**), Mac3 (red, **b**) and F4/80 (green, **c**) staining performed in nude and *mdx* muscles. Magnification areas (right panels) show details of staining (black arrows), from rectangles. Dashed yellow lines indicate the borders between muscle and scaffold tissues. Nuclei are stained in blue with Hoechst. Scale bars: left panel **a** = 100 µm; right panel **a** = 20 µm; left panel **c** = 100 µm; right panel **c** = 20 µm.

Data reported in the literature show that the local inflammation occurring after implantation of biomaterials promotes neovascularisation and tissue integration [34]. Analysis of inflammatory cells in long-term P73-implanted tissues showed a high uptake of esterase staining in the scaffolds from both nude and *mdx* mice (Figure 8a). Since esterase is a nonspecific histochemical stain used to identify macrophage cells (Figure 8a), lysosomes, and neuromuscular junctions (Supplementary Figure S4a), we analysed other inflammatory markers such as Mac3 and F4/80 (Figure 8b,c). Mac3, a marker of activated monocyte/macrophage cells [35], is highly expressed in P73 scaffolds implanted in muscles from both nude and *mdx* mice (Figure 8b). Similar expression pattern was observed for F4/80, a marker of mature macrophages, in P73 scaffolds from implanted muscles of nude and *mdx* mice (Figure 8c). Overall, these data confirmed that the majority of recruited/resident inflammatory cells populating the implanted-scaffolds during acute and chronic muscle wasting conditions are macrophages. However, immunofluorescence analysis of mannose receptor and F4/80 expression do not reveal the presence of macrophages in the scaffold of not-injured implanted muscles from C57BL/6 wt healthy mice (Supplementary Figure S5). This data confirmed the biocompatibility of P73 scaffold and suggest that the inflammatory events occurred after its implantation in nude and *mdx* mice were strictly related to the degenerative condition (Figure 8).

## 3. Discussion

A suitable muscle graft substitute able to instruct the in vivo environment to form muscle is an emerging challenge in muscle tissue engineering. Skeletal muscle consists of bundles of highly oriented muscle fibres in an extracellular matrix to form an organised syncytial tissue with high nuclear density [36]. The parallel orientation of muscle fibres guarantees the generation of longitudinal force after contraction that is induced by motoneuron activity in vivo [37]. It has been extensively demonstrated that a complexity of signals guides the formation of functional skeletal muscle. For instance, cell proliferation and differentiation are intimately depending on the chemical, mechanical and topography of the substrate (phenomenon called “contact guidance”). In light of these crucial aspects, ideal scaffolds for skeletal muscle regeneration must exhibit specific properties: (i) the topography of the highly-ordered matrix fibre; and (ii) elastic structure for myotubes contraction.

Here, we introduce a promising new matrix, i.e., P(BCE-*co*-TECE) fibre mats, for skeletal muscle tissue regeneration. In particular, the effect of the chemical composition, i.e., the amount of PEG-like co-units, and of the topography (i.e., fibrous vs. flat surfaces and fibre dimension) on cell attachment, proliferation, and differentiation has been studied with the aim of finding the best combination between physico-mechanical properties and biocompatibility. Indeed, it has been recently demonstrated that presence of ether-oxygen atoms along poly(butylene succinate) backbone had a beneficial effect on the promotion of cell attachment and proliferation [33]. In this view, P(BCE-*co*-TECE) copolymers have been fully characterised and subjected to electrospinning to obtain oriented micro and sub-microfibrous scaffolds that were characterised in their morphological, mechanical, and wettability properties. Hydrolysis rate under physiological conditions was investigated, too.

The tensile properties of the analysed polymers can be explained based on the degree of crystallinity. Indeed, PBCE, the most rigid material, displayed the highest amount of crystal phase (PBCE χ**_c_** = 86%), while P(BCE-*co*-TECE) copolymers had a lower amount of rigid crystal dispersed in a higher amount of mobile amorphous phase (P82 χ**_c_** = 74%; P73 χ**_c_** = 55%). The elastic modulus reduction of the electrospun mats with respect to the corresponding films is related to a further decrease of the degree of crystallinity and to their high porosity, which led to satisfactory mechanical properties for the intended application. Indeed, while traditional degradable polyesters exhibit elastic modulus (E) in the order of magnitude of GPa (compatible for tissue engineering of hard tissues), PBCE-based polyesters show E values of few hundreds of MPa and their scaffold counterparts exhibit E in the range 10–50 MPa (Supplementary Table S4), better matching the range of elasticity of soft tissues such as skeletal muscle.

The effects of physicochemical features of the scaffolds surface (i.e., chemical composition, energy, stiffness, wettability, charge, and roughness topography) in shaping cell adhesion, proliferation and commitment into specific lineage differentiation has been reported by numerous studies [38,39,40]. While is not completely understood the molecular mechanisms linking the cell “fate and the material cues”, it is clear that the cells respond to these cues in a wide array of ways, which depend upon many factors, including cell type and physicochemical properties of the substrate [39,41]. However, as reviewed by Bacakova and colleagues [42,43], some reactions to these cues are common and irrespective of material type and thus easily to be expected. For instance, the presence of oxygen-containing groups in the biomaterial surfaces led to an enhancement of the energy, polarity and wettability of the material surface, thus improving the adhesion and proliferation of the cells on this surface [33,42]. Beside surface chemistry, the interaction of cells with nanoscale/microscale topography has proven to be an important signalling factor in modulating complex cellular processes such as cell polarisation, proliferation and differentiation [44].

The chemical composition of the material surface is another important factor that, by influencing other physicochemical properties of the surface, can in turn modify the character of the cell–surface interaction. We observed that the incorporation of different amounts of ether-oxygen atoms along PBCE macromolecular chain confers to these new electrospun materials more favourable properties (i.e., lower stiffness, higher hydrophilicity, and a higher amount of anchoring points for cells in comparison to PBCE) for myogenic cells adhesion and growth. We found that C2C12 myoblasts cultured on surface with the highest TECE content (P73) showed a greater proliferation rate, proving the role of matrix stiffness in mediating cell response [39,40,45]. Cell adhesion is also determined by surface wettability [42]. Our results showed that the presence of oxygen-containing groups increased the polar component of the polymer surface, making this surface more hydrophilic and thus more suitable for cell adhesion.

Topography of materials is one of the key factor that can be harnessed to modulate cell behaviour [44,46]. Consistent with previous studies, we confirmed that cell adhesion and arrangement depend on structural and morphological guidance from the underlying substrate [47,48]. While parallel and oriented micro and sub-microfibres affect the myoblast cytoskeleton organisation and alignment, P(BCE-*co*-TECE) films leads to a chaotic pattern of cell distribution.

Consistent with previous reports [49,50], we observed that the inherent alignment within the electrospun forcing the cell elongation guide the myoblasts towards the activation of the myogenic process in both proliferation and differentiation media. We found that MyoD, the earliest marker of myogenic commitment [51], and myogenin, a late transcription factor involved in the skeletal muscle development [52], showed a higher expression in cells cultured on micro and sub-microfibres compared to film counterparts. Similar results were also obtained for late myogenic genes, such as MyHC and M-Cadherin, further suggesting that P(BCE-*co*-TECE) fibrous scaffolds were suitable materials for muscle regeneration.

Interestingly, we also reported that in the richest TECE-containing polymer (P73), the fibre dimension is a decisive cue in influencing the complex process of myogenic differentiation. Increasing the fibre dimension of a 2-fold factor (from around 450 to around 900 nm), a higher activation of both early (MyoD) and late-stage myogenic differentiation genes (myogenin, MyHC and M-cadherin) was obtained. A possible explanation for this finding may be related to the extent and the strength of cell adhesion. While further investigations need to be performed to gain deeper insight into material cytotoxicity [53], it possible to hypothesise that the microstructures drive the cells to the critical degree of cytoskeleton-tension and focal adhesion numbers. These structural modifications force C2C12 cells to activate the myogenic pathways in a more efficient way in comparison with sub-micro surfaces.

The key role of substrate stiffness in myogenesis is also demonstrated by the evidence that the early myogenesis (i.e., MyoD nuclear expression) is delayed on very stiff substrates (film), while the late myogenesis (i.e., myotubes formation) can only occur on elastic electrospun surfaces. We then performed in vivo implantation experiments by using P73-micro because, among the P(BCE-*co*-TECE) electrospun in vitro tested substrates, it exhibited the best structural characteristics and cellular compliance. In particular, histological analysis of long term P73 implantation in TA muscles (from both regenerating and dystrophic muscles) showed a surprising ability of such scaffold to integrate on the surface of host muscle tissue. Six weeks afterP73 implantation, scaffolds appeared highly cellularised in both regenerating and dystrophic muscle models. Analysis of recruited/resident cells in the implanted areas (comprised between the scaffold and the covered muscle regions) showed that the majority of cells populating the P73 are inflammatory cells. Esterase, Mac3, F4/80, and mannose receptor staining also revealed that macrophages predominantly colonised the implanted scaffold, while a minor part of the heterogeneous cell population include endothelial cells forming vessels within the tissues. The presence of neovasculature confirmed both the success of muscle graft [54], and the strong intimate connection between macrophages and endothelial cells in vasculogenesis of implanted tissues, as described for other engineering scaffolds [54] and during muscle regeneration [55,56].

Interestingly, in our model we did not observe any of the deleterious effects of the immune infiltrate on the scaffold, as already reported for other biomaterials, (e.g., rejection and/or structural alterations). Besides immunocompetent mice, we also used immunocompromised mice, which lack immune T cells, and are considered useful models for future allogeneic cell-based strategies in muscle engineering studies. Taken together, our data showed that P73 electrospun substrate is a promising candidate for further in vivo studies exploring drug and cell delivery in muscle degenerative conditions.

## 4. Materials and Methods

### 4.1. Polymers

Poly(butylene 1,4-cyclohexanedicarboxylate) homopolymer (PBCE, Figure 1a) and two random copolymers, made of butylenecyclohexanedicarboxylate (BCE) and triethylenecyclohexanedicarboxylate (TECE) co-units (Figure 1b), with different molar compositions were synthesized as previously described [32]. In particular, P(BCE80-*co*-TECE20) was labelled P82 and P(BCE70-*co*-TECE30) was labelled P73.

#### 4.1.1. Preparation of PBCE and P(BCE-*co*-TECE) Substrates

PBCE and P(BCE-*co*-TECE) electrospun scaffolds were produced by an in-house electrospinning apparatus: it consists of a high voltage power supply (Spellman, Hauppauge, NY, USA, SL 50 P 10/CE/230), a syringe pump (KDScientific 200 series), a glass syringe, a stainless-steel blunt-ended needle (inner diameter = 0.84 mm) connected with the power supply electrode and a grounded high-speed rotating collector (tangential speed 16.2 m/s). The polymer solution was dispensed, through a Teflon tube, to the needle vertically placed on the collecting mandrel. The electrospinning process was carried out at room temperature (RT) and relative humidity RH = 40 ÷ 50%. By varying electrospinning conditions, we were able to produce micrometric and nanometric fibres for each polymer. Six types of scaffolds were thus produced: micro-PBCE and sub-micro-PBCE; micro-P82 and sub-micro-P82; and micro-P73 and sub-micro-P73 (Supplementary Table S1). Before detaching the scaffolds from the rotating collector, they were wetted in absolute ethanol. This constrained ethanol treatment enabled chain relaxation to occur at the molecular level but prevented scaffold shrinkage and changes of fibre morphology [57]. Films of PBCE homopolymer and of P(BCE-*co*-TECE) copolymers were obtained by compression moulding the polymers between Teflon plates, with an appropriate spacer, at a temperature *T* equal to *T_m_* + 40 °C, for 2 min under a pressure of 2 ton/m^2^. The obtained films (0.2 mm thick) were labelled as film-PBCE, film-P73 and film-P82.

#### 4.1.2. Material Characterisation

Scanning electron microscope (SEM) observations of electrospun scaffolds were carried out using a Philips 515 SEM (FEI, Eindhoven, The Netherlands) at an accelerating voltage of 15 kV on samples sputter-coated with gold. The distribution of fibre diameters was determined through the measurement of about 200 fibres by means of an acquisition and image analysis software (EDAX Genesis, Eindhoven, The Netherlands). Static contact angle measurements were performed on polymer films by using a KSV CAM101 instrument at ambient conditions by recording the side profiles of deionised water drops for image analysis. Five drops were observed on different area for each film and contact angles were reported as the average value ± standard deviation.

Stress–strain measurements were performed with an Instron 4465 tensile testing machine on rectangular films and on rectangular sheets cut from electrospun mats.

Hydrolytic degradation experiments were performed by incubating polymeric films in phosphate buffered solution (0.1 M, pH = 7.4) and 37 °C in a shaking water bath (SW22 Julabo, Seelbach, Germany). The buffer solution was periodically changed to keep the pH constant. At different time intervals, triplicate specimens for each sample were recovered from the bath, repeatedly washed with deionised water, and dried over P_2_O_5_ under vacuum for 2 days to reach constant weight. Loss of mass was determined by comparing the dry weight remaining at a specific time with the initial sample weight. Molecular weight data were obtained by gel-permeation chromatography (GPC) at 30 °C using a 1100 Hewlett Packard system equipped with PLgel 5 µm MiniMIX-C column (250/4.6 length/i.d., in mm). A refractive index was employed as a detector and chloroform was used as eluent with a 0.3 mL/min flow. Sample concentrations of about 2 mg/mL and polystyrene standards were used.

### 4.2. In Vitro Studies of Myogenic Potential

#### 4.2.1. Cell Cultures

Murine myoblast cell line C2C12 (ATCC CRL-1772) was cultured in proliferation medium (PM) constituted of Dulbecco’s modified Eagle medium (DMEM, Sigma-Aldrich, St. Louis, MO, USA) supplemented with 10% foetal bovine serum (FBS), 1% penicillin/streptomycin, 1% l-glutamine, and 1% sodium pyruvate (Thermo Fisher Scientific, Paisley, Scotland, UK). Cells were cultured, routinely trypsinised after confluence, and maintained in incubator at 37 °C with a 5% CO_2_ atmosphere.

For both proliferation and differentiation studies, before cell seeding, scaffolds were cut in round shaped pieces (2 cm^2^ area, about 80 µm thick), assembled in 24-well CellCrown supports (Scaffdex, Tampere, Finland) in order to prevent them floating in the cell culture medium, and inserted into the wells of 24-well plates. Scaffolds were sterilised with EtOH 90% then treated with EtOH 70% for 15 min, washed twice in phosphate-buffered saline (PBS) for 10 min, assembled in a 24-well plate, and incubated overnight in PBS containing 1% penicillin/streptomycin under UV light. Sterile scaffolds were incubated with serum-free culture medium (DMEM) at 37 °C for 2 h before cell seeding [58,59,60].

#### 4.2.2. Cell Proliferation Studies

C2C12 cells were cultured on sterilised scaffolds: (i) film-, micro-, sub-micro-PBCE; (ii) film-, micro-, sub-micro-P82; (iii) film-, micro-, sub-micro-P73; and (iv) plastic tissue culture plates (plastic), used as internal control. Briefly, on day 0 a drop of cell suspension containing 1.0 × 10^5^ cells was added onto the top of each scaffolds. After 30 min, 1 mL of cell culture medium was added to each well and changed every 2 days.

##### Viability Assay

Cell viability on different scaffolds, was evaluated on day 1 and day 7 using the quantitative CCK8 test (Cell Counting Kit-8, Sigma-Aldrich) according to manufacturer’s instructions. Aliquots of 100 µL were sampled, and the absorbance values were measured at 460 nm by a microplate reader (BioRad Laboratories, Hercules, CA, USA). A standard curve of cell viability was used to express the results as cell proliferation rate, defined as the increase in the ratio of cell number at days 1 and 7 over the seeded cell number on day zero (1.0 × 10^5^ cells/scaffold).

##### Morphological Analysis

For morphological study, cells were seeded at a final density of 5 × 10^4^/cm^2^ in PM onto the top of all different samples and then observed under Confocal Laser Scanning Microscope (CLSM) and Scanning Electron Microscopy analysis (SEM).

Specifically, after 24 h of culture, cells were washed with PBS, fixed with 4% (*w*/*v*) paraformaldehyde solution (PFA) for 30 min at 4 °C and permeabilised with 0.1% Triton X-100 for 5 min. For focal adhesion detection, cells were incubated with the primary mouse anti-α vinculin antibody (1:500 in 1% Bovine serum Albumin, BSA, Sigma-Aldrich) overnight at 4 °C, then with Alexa-Fluor-488 conjugated secondary antibody (1:500 in 1% BSA, Invitrogen, Carlsbad, CA, USA) for 1 h at Room Temperature (RT). In order to visualise the f-actin cytoskeleton organisation, cells were then stained with Tetramethylrhodamine B isothiocyanate (TRITC) phalloidin conjugate solution (10 μg/mL, EX/EM maxima ~540/575, Sigma-Aldrich) in PBS for 40 min at RT. Nuclei were counterstained with Hoechst 33342 (2 μg/mL) for 5 min. Samples were observed with a confocal fluorescence microscope (Leica TCS SPII Microsystems, Leica Microsystems, Bensheim, Germany).

For SEM analysis C2C12 were seeded on the different scaffolds and treated as previously described [61]. The specimens were sputter coated with gold and observed at 3000× magnification with a Leica Cambridge Stereoscan 440 microscope (Leica Microsystems, Bensheim, Germany) at 8 kV.

#### 4.2.3. Cell Differentiation Studies

Cell differentiation analysis was carried out in order to assess P73 scaffold capability of supporting cell fusion and myotubes formation. Twenty-four hours after seeding, C2C12 were maintained in PM or differentiation medium (DM), containing 2% of Horse Serum (HS) for 7 or 14 days of culture, replacing the culture medium every 2 days.

##### Real-Time RT-PCR (RT-qPCR)

At 7 and 14 days of culture, total RNA was extracted from cells seeded on P73 scaffold using Trizol reagent, according to the manufacturer’s protocol (Invitrogen). The subsequent reverse transcription was performed using the iScript™ cDNA (Bio-Rad, Hercules, CA, USA). Quantitative RT-PCR analysis was performed in a 48-well optical reaction plate using a MiniOptico^®^ Real Time PCR System (BioRad). Gene expression was analysed in triplicate and normalised to the CT mean of phosphoglycerate kinase (PGK) using the Livak method [62]. Primers used are listed in Supplementary Table S2 (Invitrogen).

##### Immunofluorescence

After 14 days of culture in PM or DM, cell-seeded P73 biomaterials were subjected to immunofluorescence staining for myosin heavy chain (MyHC). Four percent PFAfixed cells were incubated with a mouse anti-MyHC (Hybridoma Bank, Iowa, IA, USA) overnight at 4 °C, following by 1 h incubation at RT with Alexa 488-conjugated anti-mouse secondary antibody (1:500 in PBS, Invitrogen, A21206). Cells nuclei were counterstained with Hoechst 33342 (2 μg/mL) and observed by confocal fluorescence microscope.

##### ELISA Assay

After 14 days of culture, the amount of sarcomeric myosin protein produced by C2C12 myotubes cultured on P73 biomaterials, either in PM or DM, was determined by an ELISA assay as previously described [63]. In brief, the samples were washed extensively with sterile PBS to remove culture medium and then incubated for 24 h at 37 °C with 0.5 mL of sterile sample buffer (20 mM Tris-HCl, 4 M GuHCl, 10 mM EDTA, 0.066% (*w*/*v*) sodium dodecyl sulphate (SDS), pH 8.0). At the end of the incubation time, after centrifugation (13,000 rpm, 15 min, 4 °C) to remove cell debris, total protein concentration was determined by a bicinchoninic acid-based assay kit (Pierce Biotechnology Inc., Rockford, IL, USA). Calibration curve to measure myosin was performed and the amount of myosin from samples was expressed as pg/(cell per scaffold).

### 4.3. In Vivo Biocompatibility Studies

#### 4.3.1. Scaffold Implantation

P73-micro scaffolds (Ø = 6 mm^2^) were implanted on the right tibialis anterior (TA) muscles of 6-week-old male wild-type C57BL/6 (Charles River, Beerse, Brussels, Belgium) mice. Six-week-old male athymic nude (NMRI, nu/nu, Charles River, MA, USA) and *scid*/*mdx* mice, congenitally lacking B and T lymphocytes [64], were also used to assess the biocompatibility of the scaffold (Supplementary Figure S2). The contralateral muscles were sham-operated and used as internal control. To study the response of the scaffold under acute muscle degeneration, TA muscles of nude mice were freeze-injured by a dry ice precooled steel probe (Ø = 6 mm^2^) applied on exposed muscle for 10 s. Mice were euthanised 4 and 6 weeks after muscular P73-micro implantation. All the in vivo experiments were performed in accordance with ARRIVE guidelines and following the three Rs rule of Replacement, Reduction, and Refinement principles [65]. Mice were treated with protocols approved by the animal experimentation ethics committee of the KU Leuven, Belgium (P057/2017, 6 Mar 2017).

#### 4.3.2. Histology and Histochemistry

TA muscles were dissected, embedded in tissue freezing medium (Leica, Wetzlar, Germany) and frozen in liquid nitrogen-cooled isopentane. Tissue cryosections (7 μm thickness) were obtained using a Thermo Scientific Cryostar, NX70 (Waltham, MA, USA). For histological analysis, the sections were stained with hematoxylin and eosin (H&E, Sigma-Aldrich), using standard methods. Esterase staining was performed as previously reported [66]. Photomicrographs were obtained using a Nikon Microscope system (Nikon Digital Sight DS 5M, Groot-Bijgaarden, Belgium) and Zeiss Axio Imager Microscope (Carl Zeiss, Oberkochen, Germany).

#### 4.3.3. Tissue Immunostaining

Cell cultures and muscle cryosections were fixed in 4% PFA for 10 min at RT. After incubation with 1% BSA, 0.2% Triton X-100 for 30 min, samples were incubated with 10% donkey serum for 30 min at RT. Primary antibodies used: mouse anti-MyHC (Hybridoma Bank) undiluted, overnight at 4 °C; rabbit anti-Laminin (SIGMA, L9393-.5ML, 1:500) in PBS, overnight at 4 °C; Isolectin Alexa Fluor 488 isolectin GS-IB4 conjugate (molecular probes, I21411, 1:300) in PBS, 30 min at RT; rabbit anti-Ki67 (Abcam, Cambridge, UK, ab15580, 1:1000) in PBS, overnight at 4 °C; rat anti-Mac-3 (BD pharmingen, Brussels, Belgium, 1:50) in PBS, overnight at 4 °C; rat F4/80 (Abcam, ab6640, 1:50) in PBS, overnight at 4 °C; rabbit anti-alpha smooth muscle actin antibody (Abcam, ab15734, 1:200) in PBS, overnight 4 °C; rabbit anti-Von Willebrand factor (Abcam, ab6994, 1:400) in PBS, overnight at 4 °C; mouse anti-mannose receptor (Abcam, ab8918, 1:300) in PBS, overnight at 4 °C.

Secondary antibodies used (1:600 in PBS): anti-mouse-Alexa 594 (Invitrogen, A21203); anti-rabbit-Alexa 488 conjugated antibodies (Invitrogen, A21206); anti-rat-Alexa 488 conjugated antibodies (Invitrogen, A21208); anti-rat-Alexa 555 conjugated antibodies (Invitrogen, A21434); anti-rabbit-Alexa 594 conjugated antibodies (Invitrogen, A21207); and anti-mouse-Alexa 488 conjugated antibodies (Invitrogen, A21202). Nuclei were stained with 0.5 mg/ml Hoechst 33342 (Thermo Scientific). Photomicrographs were obtained using a Nikon microscope system (Nikon Eclipse Ti) equipped with a Qimaging fast 1394 Camera (QICAM 12-bit 21609), 1392 × 1040 pixels resolution, with the Image-Pro software (version 6.3, Media Cybernetics, Abingdon Oxon, UK).

### 4.4. Statistical Analysis

Each experimental condition was performed in triplicate and at least in three independent experiments. All results are expressed as mean ± SD. Differences between groups were tested by two-way ANOVA. Bonferroni post-hoc test was used to correct for multiple comparisons. The criteria for statistical significance were * *p* < 0.05. All calculations were generated using GraphPad (GraphPad Inc., San Diego, CA, USA).

## 5. Conclusions

In this contribution we showed, for the first time, the use of electrospun PBCE-based scaffolds in skeletal muscle tissue engineering. In vitro analyses revealed that the copolymerisation of BCE units with TECE co-units effectively modifies some important materials’ features. The presence of oxygen-containing functional groups contributes to increase the surface wettability and to decrease the scaffold stiffness to obtain suitable constructs for myogenic cell adhesion and proliferation. Furthermore, despite having the same chemical composition, the P73 microsurface accelerates the activation of the myogenic program in the cells in comparison with the sub-microform. Promising results were also obtained from in vivo studies, showing a safe and remarkable histological interaction between host tissue and implanted-biomaterial.

In conclusion, our results showed that electrospun PBCE-based scaffolds provide substrates mimicking the extracellular matrix cues useful for the generation of tissue-engineered muscle.

## Figures and Tables

**Table 1:** Experimental design.

***In Vitro Studies of Myogenic Potential***			
**Proliferation Assays**	**Sample**	**days**	**Medium**
Cell Viability	PBCE, P82 and 73 (film, micro- and sub-micro)	1–7	PM
Immunofluorescence		1	
SEM analysis		7	
**Differentiation Assays**	**Sample**	**days**	**Medium**
Real-Time PCR	P73 (film, micro- and sub-micro)	7–14	DM
Immunofluorescence		14	
Enzyme-linked immunosorbent assay (ELISA)		14	
***In Vivo Biocompatibility***			
***In vivo* Biocompatibility**	**Sample**	**weeks**	**Murine Model**
Scaffold Implantation	P73 (micro)	4/6	Wild type C57BL/6 Nude and *scid*/*mdx*
Histology & Histochemistry			
Tissue Immunostaining			

PBCE = poly(butylene1,4-cyclohexandicarboxylate; P82 = P80/20; P73 = P70/30; PM = Proliferation Medium; DM = Differentiation Medium.

## Data Availability

The raw/processed data required to reproduce these findings cannot be shared at this time due to technical or time limitations.

## Supplementary Materials

Supplementary materials can be found at http://www.mdpi.com/1422-0067/19/10/3212/s1.
