# Supplementary material for: Ether-Oxygen Containing Electrospun Microfibrous and Sub-Microfibrous Scaffolds Based on Poly(butylene 1,4-cyclohexanedicarboxylate) for Skeletal Muscle Tissue Engineering
Source: Int J Mol Sci. 2018 Oct 17;19(10):3212. doi: 10.3390/ijms19103212 (PMC6214009; doi:10.3390/ijms19103212)
Supplement: Supplementary file 1 [file ijms-19-03212-s001.pdf]

Table S1. Electrospinning solutions and process parameters

sample	solution for electrospinning	process parameters[a]
micro-PBCE	30 w/v% in TFE[b]	d=20 cm, r=1.2 ml/h, ΔV=15 kV
sub-micro-PBCE	20 w/v% in TFE	d=20 cm, r=1.2 ml/h, ΔV=14 kV
micro-P82	22 w/v% in TFE	d=20 cm, r=1.2 ml/h, ΔV=13 kV
sub-micro-P82	20 w/v% in TFE/DMAC[c]=90/10 (v/v)	d=20 cm, r=0.6 ml/h, ΔV=13 kV
micro-P73	30 w/v% in TFE	d=15 cm, r=2.4 ml/h, ΔV=14 kV
sub-micro-P73	22 w/v% in TFE	d=20 cm, r=0.6 ml/h, ΔV=18 kV

a) d= needle-to-collector distance; r= solution flow rate; ΔV= applied voltage.
b) TFE= 2,2,2-Trifluoroethanol, purchased from Sigma-Aldrich.
c) DMAC= N,N-Dimethylacetamide, purchased from Sigma-Aldrich.

Table S2. Primers used for qRT-PCR.

Genes	Accession number	Annealin g T°	Forward 5'-3'	Reverse 3'-5'	Amplicon size (bp)
PGK[a)]	NM_008828.3	60°C	CAAAATGTCGTCTTCCAACAAG	AACGTTGAAGTCCACCCTCAT	115
MyoD	NM_010866.2	60°C	TACAGTGGCGACTCAGATGC	TAGTAGGCGGTGTCGTAGCC	116
Myog[b)]	NM_031189.2	58°C	GGGCCCCTGGAAGAAAAG	AGGAGGCGCTGTGGGAGTT	363
MyHC	NM_007710.2	60°C	CCTGTTTGATCCCATCATCC	AGCACATAGTTGGGGTCCAG	119
M-cadh[c)]	NM_007662.2	60°C	CTTGGGTGCCACGGATGA	ATGCAGGCCCTCGGAGAC	160

[a)] PGK, phosphoglycerate kinase; [b)] Myog, myogenin; [c)] M-cadherin.

Table S3. Calorimetric data of PBCE and P(BCE-*co*-TECE) copolymers in form of films and electrospun scaffolds from first heating scan (heating rate 20°C/min).

Polymer	T_m (°C)	$\Delta H_m{}^a$(J/g)	χ_c (%)^b
film-PBCE	47-155-165^c	67	86
micro-PBCE	58-170^c	48	62
sub-micro-PBCE	58-166^c	42	54
film-P82	43-130-135^c	58	74
micro-P82	55-133^c	40	51
nano-P82	50-133^c	36	46
film-P73	43-110^c	43	55
micro-P73	51-113^c	32	41
nano-P73	54-111^c	29	37

a) neat ΔH_m
b) crystallinity degree from Equation 1
c) multiple melting peaks

Differential Scanning Calorimetry (DSC)

Differential Scanning Calorimetry (DSC) measurements were carried out using a TA Instruments Q100 DSC equipped with the Liquid Nitrogen Cooling System (LNCS) accessory. DSC scans were performed from –50°C to 200°C in helium atmosphere. A rate of 20°C/min was used during heating scans whereas the cooling scans were performed at a rate of 10°C/min. The degree of crystallinity (χ_c) was calculated by using equation 1:

$$\chi_c = \frac{\Delta H_m}{\Delta H_m^0} \cdot 100 \qquad [1]$$

Where ΔH_m is the malting enthalpy associated to the first heating scan and $\Delta H_m{}^0$ is the theoretical melting enthalpy of the 100% crystalline PBCE homopolymer, equal to 78 J/g.[1]

Table S4. Mechanical data of PBCE and P(BCE-*co*-TECE) copolymers in form of films and electrospun scaffolds

**Sample**	**E (MPa)**[a]	**σ_b (MPa)**[b]	**ε_b (%)**[c]
film-PBCE	466 ± 8	35 ± 2	25 ± 7
micro-PBCE	54 ± 5	20 ± 3	54 ± 5
sub-micro-PBCE	58 ± 7	16 ± 2	74 ± 9
film-P82	270 ± 9	18 ± 2	221 ± 8
micro-P82	34 ± 2	14 ± 1	64 ± 8
sub-micro-P82	37 ± 4	11 ± 1	43 ± 2
film-P73	159 ± 3	15 ± 1	440 ± 40
micro-P73	14 ± 2	8.3 ± 0.8	51 ± 4
sub-micro-P73	16 ± 2	8 ± 1	44 ± 2

E = tensile elastic modulus b) σ_b = stress at break c) ε_b = elongation at break

Figure S1

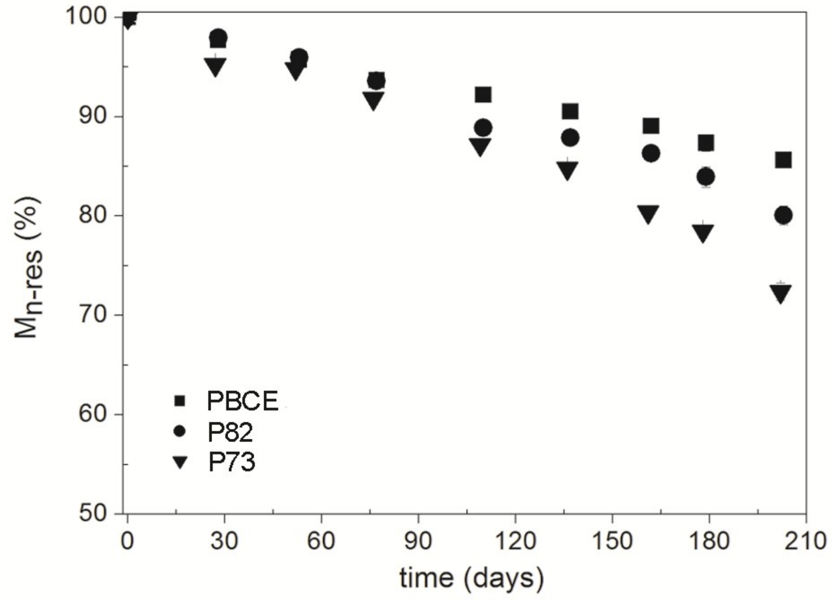

S1. **Polymer hydrolytic degradation.** Percentage of residual number average molecular weight ($M_{n\text{-}res}$%) as a function of degradation time for PBCE and P(BCE-*co*-TECE) copolymers in form of films.

Figure S2.

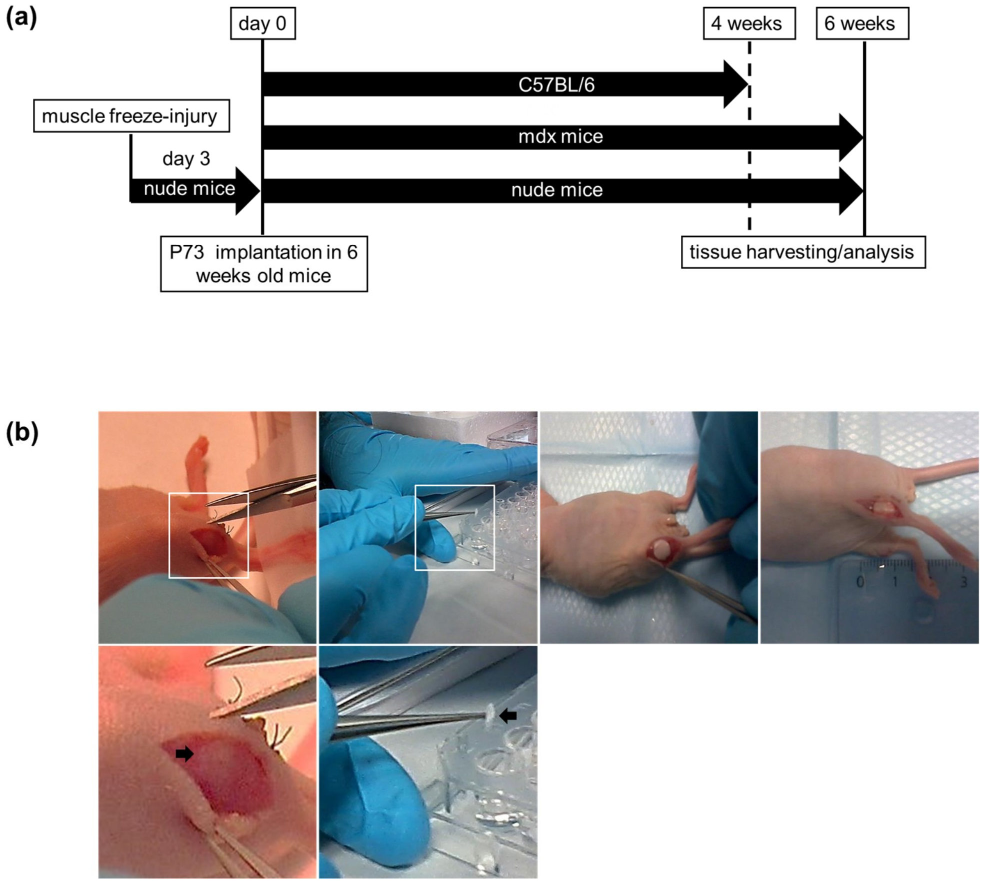

S2. (a) *In vivo* experimental design. (b) Details of scaffold implantation in injured *tibialis anterior* (TA) muscle of athymic nude mouse. Lower panels show magnification areas of freeze-injured TA (left panel) and P73 scaffold (right panel).

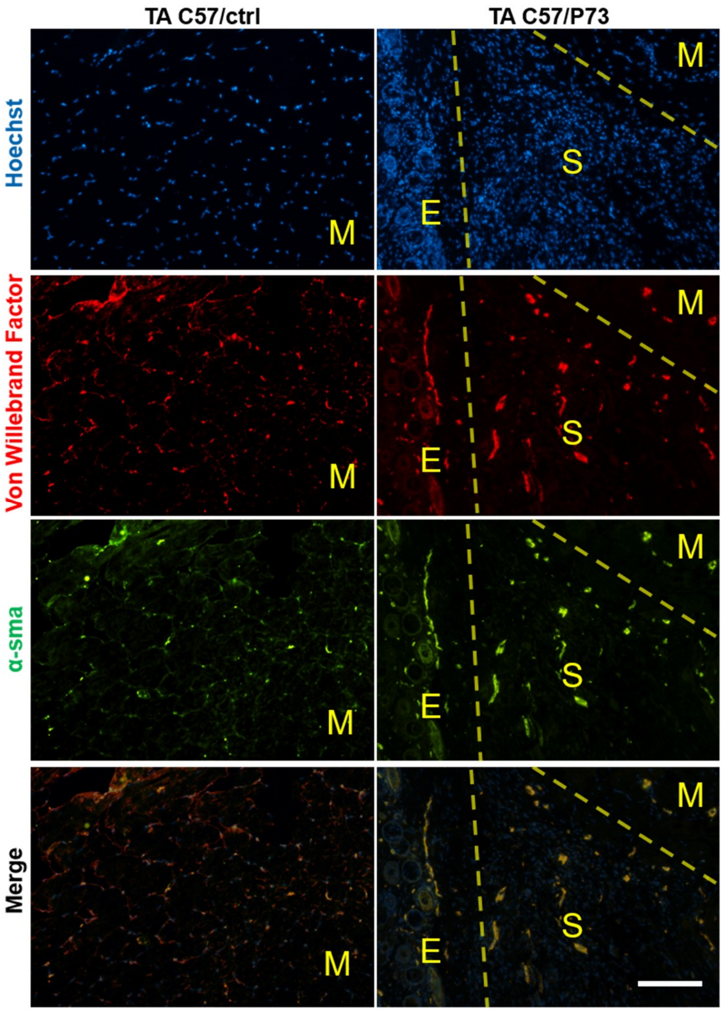

Figure S3

S3. Immunofluorescence analysis of Von Willebrand factor (red) and α-sma (α-smooth muscle actin in green) in *tibialis anterior* (TA) muscles from C57BL/6 healthy and not injured P73-implanted mice. Nuclei are stained in blue with Hoechst. S = scaffold; M = muscle; E = epidermis. Scale bar = 100 μm.

Figure S4

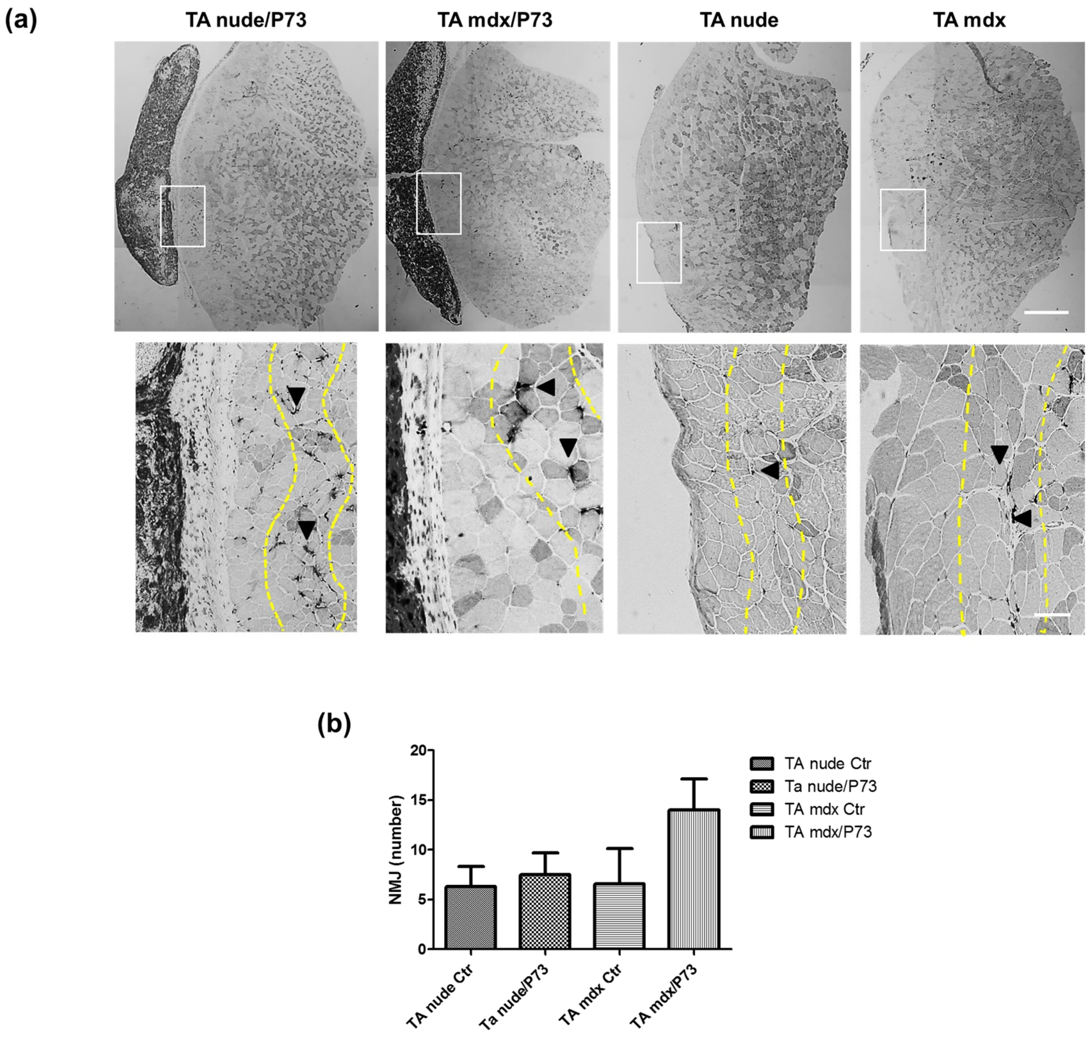

S4. Histological analysis of neuromuscular junction content in P73-implanted muscles. (a) P73-implanted and control muscles sections of athymic and mdx mice stained with esterase showing neuromuscular junctions (black arrowheads) near to the transplanted area (dashed yellow lines). Bottom panels show magnification areas from rectangles. (b) Quantification of neuromuscular junctions (NMJ) from (a) (n = 3 each experimental condition). Scale bars: upper panel = 0.5 mm; lower panel = 200 µm.

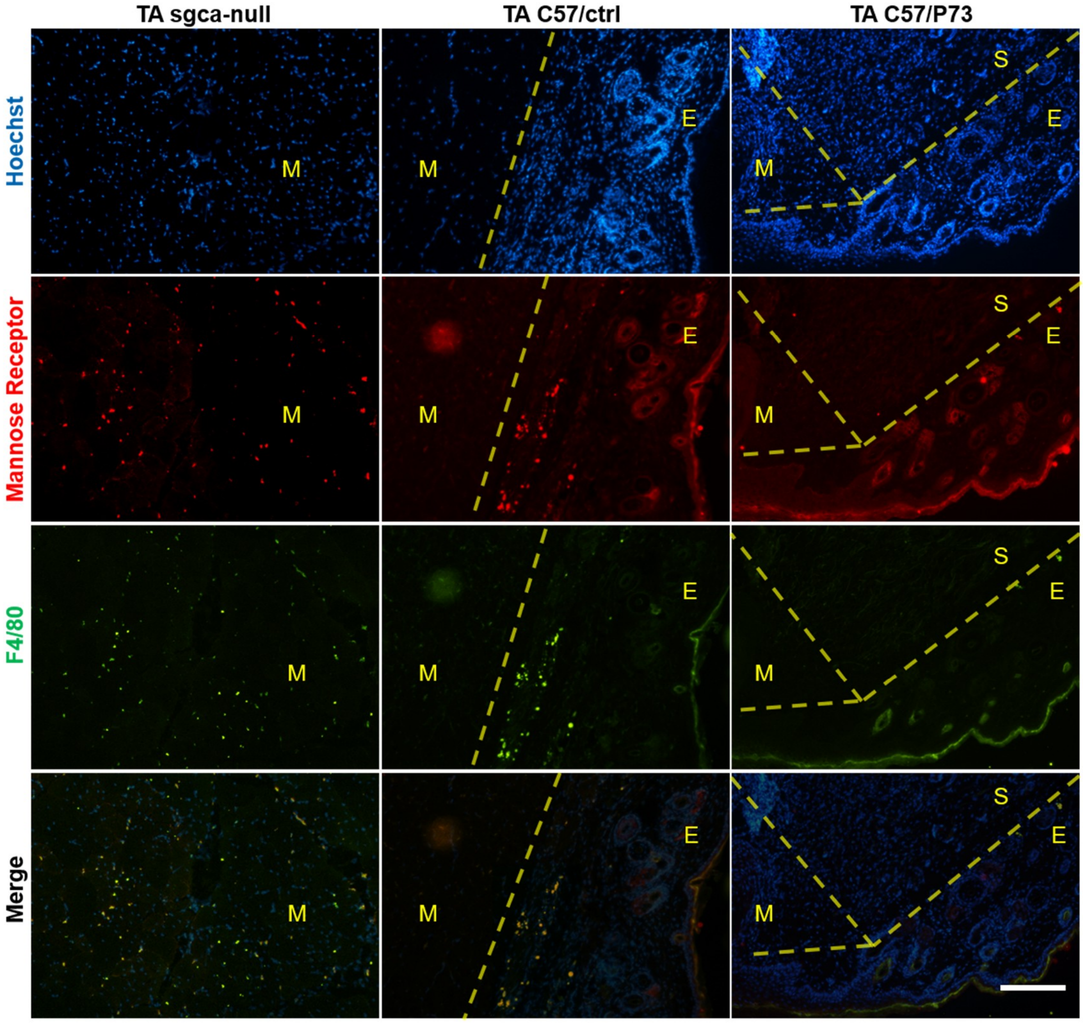

Figure S5

S5. Immunofluorescence analysis of mannose receptor (red) and F4/80 expression (green) in *tibialis anterior* (TA) muscles from Sgca-null, C57 not-implanted (ctr) and not injured C57/P73-implanted mice. Nuclei are stained in blue with Hoechst. S = scaffold; M = muscle; E = epidermis. Scale bar = 100 µm.

Reference

(1) Celli, A.; Marchese, P.; Sullalti, S.; Berti, C.; Barbiroli, G. Eco-Friendly Poly(butylene 1,4-Cyclohexanedicarboxylate): Relationships Between Stereochemistry and Crystallization Behavior. *Macromol. Chem. Phys.* **2011**, *212* (14), 1524–1534.
